# Development of a Polypropylene-Based Material with Flame-Retardant Properties for 3D Printing

**DOI:** 10.3390/polym16060858

**Published:** 2024-03-21

**Authors:** Eleonora Lorenzi, Rossella Arrigo, Alberto Frache

**Affiliations:** 1 Department of Applied Science and Technology, Politecnico di Torino, Viale Teresa Michel 5, 15121 Alessandria, Italy; eleonora.lorenzi@polito.it (E.L.); rossella.arrigo@polito.it (R.A.); 2 Local INSTM Unit, 15121 Alessandria, Italy

**Keywords:** 3D printing, polypropylene, flame retardant, nanoclays, nanocomposite, cone calorimeter

## Abstract

In this study, a nanocomposite based on a heterophasic polypropylene copolymer containing 5 wt% of nanoclays and 3 wt% of compatibilizer was formulated via melt compounding to obtain a material suitable for Fused Filament Fabrication (FFF) processing with enhanced flame-retardant properties. From rheological analyses, the nanocomposite showed an important increase in the non-Newtonian behavior, and, therefore, improved FFF printability compared to the pristine PP COPO. A filament with suitable characteristics for FFF was produced using a single-screw extruder and subsequently 3D printed. Finally, cone calorimeter and UL94 tests were carried out on both 3D-printed and compression-molded specimens. The obtained results showed that the 3D-printed samples exhibited even better flame-retardant properties than the compression-molded ones, thus demonstrating not only the possibility of successfully developing and using functionalized PP-based filaments in 3D printing but also the possibility of obtaining enhanced flame-retardant properties compared to conventional compression molding.

## 1. Introduction

Additive manufacturing (AM) techniques [1] emerged around the 1980s and since their invention they have undergone great development and are increasingly used in industries both for prototype development and the production of functional and high-performance parts used in many areas such as the biomedical [2,3], automotive [4,5], aerospace [6] and electronics [7] sectors.

The main advantage of AM is the mold-less fabrication of parts directly from a computer-aided design (CAD), which allows the production of objects with complex geometries and multiple material combinations while properly managing materials, resulting in less waste and various other advantages over conventional manufacturing (i.e., compression molding, injection molding, etc.) [8].

Among the different 3D-printing techniques for thermoplastic polymers, Fused Filament Fabrication (FFF) [9,10,11,12] is one of the most used. This method belongs to the material extrusion additive manufacturing processes. In fact, in FFF, a molten thermoplastic filament is extruded through a movable and heated nozzle; then, is deposited on a printing platform and the desired object is constructed following a layer-by-layer approach.

Despite the rapid development of FFF techniques, their application is still limited by the modest availability of suitable materials. The polymers most widely used as feedstocks for the formulation of filaments for 3D printing are amorphous or low-crystalline polymers, like acrylonitrile butadiene styrene copolymer (ABS) and polylactic acid (PLA), and the majority of these filaments that are available on the market have no specific functionalization [13].

Polypropylene (PP) is one of the most studied and commercialized polymers, owing to its versatility given by its excellent mechanical properties, thermal stability, chemical inertness and cheapness. Nevertheless, its use in FFF techniques is still very limited and challenging [14,15,16].

Due to its semi-crystalline nature, PP is characterized by a high volumetric shrinkage during solidification, leading to warpage and delamination, and thus detachment of the printed object from the printing platform [17]. The volumetric contraction represents not only an issue during the printing step but also during the production of the filament. In fact, an ideal filament for 3D printing should have a circular section with constant diameter, but non-uniform shrinkage can lead to having an oval section. Additionally, FFF processing is strongly influenced by the rheological behavior of the material. Different studies have demonstrated that an ideal material for FFF should have non-Newtonian behavior [18,19]. In fact, low viscosity values at high shear rates, which is the presence of shear thinning, ensure a good flowability of the material during the extrusion through the nozzle, while a rapid increase in the viscosity at quasi zero-shear conditions allows the material to retain its shape during and after the deposition and to avoid oozing phenomena. Conversely, polypropylene exhibits typical Newtonian behavior, with a large Newtonian plateau at low shear rates. Finally, PP shows very low adhesion to any surface that is not PP itself, so the use of printing platforms that are typically used for PLA or ABS leads to problems of detachment of the object during the printing process [20].

All these issues cause acute inaccuracy or even failure in printing; therefore, in recent years, several research studies have focused on improving the printability of PP by decreasing the crystallinity of the polymer. This can be achieved through different strategies such as the addition of fillers (such as, carbon fibers [21], glass fibers [22,23], clay [24], talc [23] or cellulose [25]) or the use of polymeric blends and of copolymers [26]. In previous works, it was demonstrated that the addition of 20 wt% of talc in a PP-PE random copolymer leads to more pronounced non-Newtonian behavior compared to neat PP, and therefore to enhanced FFF printability. Moreover, the effect of different micro-sized (talc, calcium carbonate and silica) and nano-sized (nanoclays) fillers on the thermal properties and rheological behavior of the PP matrix were investigated [27,28]. It was demonstrated that it is possible to achieve modulable mechanical characteristics as a function of the type of embedded filler, allowing for the production of 3D-printed materials with specific characteristics.

Regarding the development of functionalized filaments for 3D printing, all studies focus on using easily printable polymers like PLA, ABS or PU. As an example, Guo et al. [29] aimed at improving the conductivity of PLA-based composites by incorporating carbonaceous fillers. In particular, the addition of 9 wt% rGO was found to be the best choice, leading to a decrease in volume electrical resistivity by nine orders of magnitude compared to the control composite. Furthermore, models and patterns printed by FDM demonstrated that the composite was suitable for printing complex shapes. Concerning the development of thermally conductive materials for 3D printing, Jiang et al. [30] studied the synergistic effect of graphene and alumina in a PLA matrix, while Liu at al. [31] developed a TPU-based composite by incorporating hexagonal boron nitride (hBN) and evaluated how the alignment of the hBN platelets, induced by the elongation flow that the material underwent during the printing process, affects the thermal conductivity properties.

One of the biggest weaknesses of many polymers is their flammability, but again the flame-retardant (FR) filaments currently available on the market or studied are always PLA- or ABS-based [32]. As an example, Guo et al. [33] developed a flame-retardant PLA-based filament using 17 wt% melamine polyphosphate (MPP) and 1 wt% Cloisite30B (C-30B). The compound was able to be extruded and fed to the 3D printer and from the UL-94 tests, both the compression-molded and 3D-printed samples achieved a V0 rating. The two samples also showed very similar behavior in the cone calorimeters test, demonstrating that the flame-retardant properties were maintained in the 3D-printed samples. Additionally, Regazzi et al. [34] explored different formulations of flame-retardant PLA with ammonium polyphosphate, melamine cyanurate and nanoclays, changing their overall content and their distribution regarding the exposed surface. The results from cone calorimeter tests showed that, compared to injection-molded samples, the FFF-printed specimens exhibited a significant decrease in the time to ignition and a slight decrease in the total heat released. This behavior has been mainly attributed to the higher sample porosity induced by the FFF process.

Therefore, it becomes apparent how, at the moment, the studies on new materials for 3D printing are divided into two categories: studies on the development of functionalized filaments for FFF, based on easily printable polymers, and studies focused on merely improving the 3D printability of PP.

The aim of this work is to combines these two themes and therefore develop a flame-retardant PP-based filament suitable for FFF processing and compare the properties of this material when processed through compression molding or 3D printing. The incorporation of nanofillers within polymer matrices has been widely exploited to improve the fire performance of polymeric materials. The biggest advantage in using nanofillers compared to other traditional flame-retardant additives is the low percentage needed (from 0.5 to 5 wt%). This is also an advantage for the 3D-printing process, as the presence of large amounts of additives could affect the proper flowability of the polymer through the printer nozzle [35,36]. The composite, based on a heterophasic PP copolymer and containing 5 wt% of organo-modified nanoclays and 3 wt% of compatibilizer, was obtained through melt compounding in a twin-screw extruder and then, after a close optimization of the process parameters, printable filaments were obtained. The thermal properties and rheological behavior of the composite were evaluated in order to assess its processability and printability. Finally, the flame-retardant properties of the compression-molded and 3D-printed samples were studied through cone calorimeter tests and horizontal UL-94 tests.

## 2. Materials and Methods

### 2.1. Materials

Polypropylene ISPLEN^®^ PB 170 G2M (hereinafter named PP COPO) from Repsol–Chemicals (Madrid, Spain) was used. This is a polypropylene–polyethylene random copolymer (density of 905 kg/m^3^ and melt flow index of 12 g/10 min (230 °C, 2.16 kg)).

The organophilic phyllosilicate Cloisite^®^20A (C20A) (density 1.80 g/cm^3^, particle size < 10 μm) was provided by BYK (Wesel, Deutschland).

To increase the compatibility between the polymer and the nanoclay C20A, polypropylene–graft–maleic anhydride (PP-g-MA) (0.6 wt% of maleic anhydride) from Sigma-Aldrich (Darmstadt, Deutschland) was used.

### 2.2. Preparation of Nanocomposite

**Figure 1:**
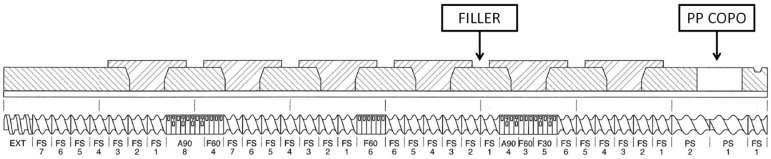
Profile of the screws used in the compounding process. It consists of different elements such as feeding elements (FS, PS), mixing elements (F30, F60, A90) and discharge elements (EXT).

The nanocomposite PP COPO + 5% C20A + 3% PP-g-MA was produced through melt compounding in a twin-screw extruder Thermo Fisher Scientific™ Process 11 (Waltham, MA, USA). The extruder has two co-rotating screws (11 mm diameter) placed inside a cylinder characterized by 7 heated zones (Figure 1). The heating temperature profile was set at 190 °C for all the zones and the screw rotation speed was set at 200 rpm. Two volumetric feeders were used: the first, for the polymer and the compatibilizer, at the beginning of the extruder; the second, for the filler, was placed about one-third through the barrel. At the exit of the extruder die, the molten polymer was cooled in a water bath and pelletized. The obtained composite was coded as PP COPO/C20A.

Samples for the rheological tests (25 mm diameter for 1 mm thickness) were obtained through a compression molding step using a hot plate press Collin P 200T (Maitenbeth, Germany). The operation was carried out by pressing the pellets at 100 bar into a right-shaped metallic mold, heated at 190 °C, for 3 min. Samples for cone colorimeter (50 × 50 × 3 mm) and UL94 (125 × 13 × 3 mm) tests were also prepared via compression molding (CM).

### 2.3. Three-Dimensional Printing Equipment

A Felfil Evo filament making machine (Torino, Italy) was used to produce filaments with a diameter of 1.75 mm. This machine is composed of a single-screw extruder, a mobile cooling fan array and a spooler with two rollers, a diameter sensor and a spool holder. The speed of the rollers is automatic and automatically self-adjusts in order to maintain the set diameter value. Processing parameters such as extrusion temperature, screw speed, power and position of the fans were optimized for each material and will be discussed in Section 3.3. Some settings, however, were kept unchanged for all of the filaments, such as a filament diameter of 1.75 mm and spooler speed of 75 rpm.

A Roboze One 3D printer (Bari, Italy), equipped with a 0.6 mm steel nozzle, was used to 3D print the cone colorimeter (50 × 50 × 3 mm) and UL94 (125 × 13 × 3 mm) test specimens. To set the parameters, the Simplify 4.1.2 3D software was used, while the Solidworks 2021 software was used in the CAD process of the various samples. As PP shows serious issues when adhering to any surface, a polypropylene 3D printer bed (Sharebot Q, Lecco, Italy) and a 3D-printing adhesive for PP (Magigoo, Curmi, Malta) were used. Some settings were kept unchanged during the optimization process for all specimens, since in previous works [27,28] they have already been proven to be good parameters for printing PP COPO-based materials, such as an infill percentage of 100%, layer thickness of 0.2 mm, bed temperature of 50 °C and extrusion speed of 50 mm/s.

### 2.4. Characterization Techniques

#### 2.4.1. Thermal Properties

The thermal properties of the formulated materials were evaluated by Differential Scanning Calorimetry (DSC) using a DSC Q20 by TA Instruments (New Castle, DE, USA). Each sample was put into a controlled chamber with nitrogen and heated from 0 °C to 250 °C at a heating rate of 10 °C/min; then, the sample was cooled at 10 °C/min back to 0 °C and finally reheated up to 250 °C with an incline of 10 °C/min.

The information achieved by this analysis include crystallization temperature (T_c_), melting temperature (Tm) and melting enthalpy (Δ*H_m_*), evaluated as the area under the exothermal peak of the heat flow during the first heating cycle. To calculate the crystallinity percentage (*X_c_*) of the PP-based pellets, the following formula was used:

(1) $$X_{c}=\frac{∆H_{m}}{\left(1-x\right)∆H_{m}^{0}}$$

where $∆H_{m}$ is the melt crystallization enthalpy, $∆H_{m}^{0}$ represents the melting enthalpy of the 100% crystalline PP and *x* is the filler weight fraction. The value of 207 J/g was considered as a reference for the 100% crystalline PP melting enthalpy [37].

#### 2.4.2. Rheological Analysis

The rheological properties of the unfilled PP COPO and PP-based composite were evaluated using an ARES (TA Instrument, USA) strain-controlled rheometer in parallel plate geometry (plate diameter = 25 mm). Preliminary strain sweep tests were carried out at 260 °C and a frequency of 10 rad/s. The complex viscosity, storage and loss moduli were measured, performing frequency scans from 100 to 0.1 rad/s. The strain amplitude was selected for each sample in order to fall in the linear viscoelastic region. To quantify the magnitude of the non-Newtonian behavior, the viscosity data were fitted with a Carreau modified model (Equation (2)) [18]:

(2) $$\eta \left(\omega \right)=\eta_{0}{\left[1+{\left(\lambda \omega \right)}^{2}\right]}^{\frac{\left(n-1\right)}{2}}+\frac{\sigma_{0}}{\omega }$$

Here, $\eta_{0}$ is the viscosity at zero-shear, λ is the relaxation time, *n* is a parameter that depends on the slope of the viscosity curve in the shear thinning zone and $\sigma_{0}$ is the yield stress.

#### 2.4.3. Morphology

The surface morphology and section of filaments were investigated using an EVO 15 Scanning Electron Microscope (SEM) from Zeiss (beam voltage: 20 kV; working distance: 8.5 mm, Oberkochen, Germany). The surfaces were investigated on small pieces of filaments by fracturing them into liquid nitrogen and then covering them with a sputtered gold layer. Quantitative compositional information was made possible through Energy dispersive X-ray Spectroscopy (EDS), which provides a spectrum with peaks related to the elements in the sample with peak amplitude proportional to the amount.

To evaluate the quality of the 3D-printed specimens, X-ray computed tomography images were obtained using the Phoenix V|tome|x S240 (Waygate Technologies, Lewistown, PA, USA) (X-ray voltage of 60 kV and current of 40 µA).

#### 2.4.4. Tensile Characterization

Tensile tests were performed using an Instron^®^ 5966 (Norwood, MA, USA) equipped with a 2 kN load cell. A crosshead speed of 1 mm/min was applied and maintained up to the achievement of 0.25% of deformation and was then increased up to 10 mm/min until breaking. The tests were carried out on five specimens and the results were averaged. The toughness of the samples was calculated as the area underneath the stress–strain curves [38].

#### 2.4.5. Flame Retardancy

To evaluate the combustion behavior, cone calorimetry tests (Fire Testing Technology, UK) were performed according to ISO 5660-1:2015 [39]. All specimens (50 × 50 × 3 mm) were subjected to a heat flux of 35 kW/m^2^, which corresponds to a temperature of 663 °C.

Finally, UL94 horizontal burning tests were carried out according to the ASTM D635-22 standard procedure on specimens of 125 × 13 × 3 mm dimensions [40].

## 3. Results

### 3.1. Rheological Characterization

In order to assess the processability and hence the FFF printability of the composite, the complex viscosity of the investigated materials was evaluated using dynamic frequency sweep tests.

**Figure 2:**
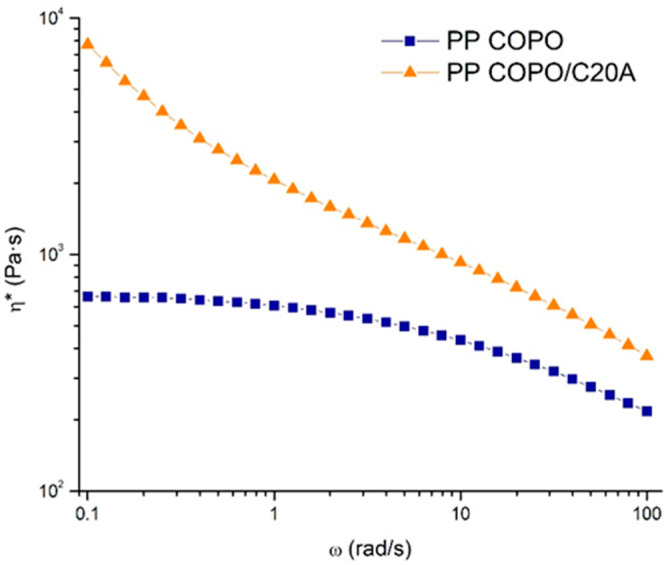
Complex viscosity curves of PP COPO and PP COPO/C20A samples.

Figure 2 reports the trends of the complex viscosity (η*) as a function of the frequency (ω) for the investigated materials. The pristine PP COPO shows marked Newtonian behavior with a Newtonian plateau developing at low and intermediate frequencies, followed by mild shear thinning at high frequencies. The introduction of the nanoclays leads instead to an important amplification of the non-Newtonian behavior of the material, with a significant increase in complex viscosity at low frequencies and a more pronounced shear thinning at high frequencies compared to the neat PP COPO [41]. The disappearance of the Newtonian plateau can be associated with the presence of embedded solid fillers which, by interacting with the polymer macromolecules, interfere with the relaxation processes of the polymer chains, hindering their complete relaxation and leading to the appearance of yield stress behavior at low frequencies [42].

Additionally, the viscosity data were fitted with the Carreau modified model (Equation (2)) [18] and the obtained fitting parameters, listed in Table 1, confirm what has already been observed by the viscosity curves. In fact, the sample PP COPO/C20A shows both higher $\eta_{0}$ and $\sigma_{0}$, indicating the presence of yield stress at low frequencies.

As already previously explained, both the presence of yield stress and shear thinning are beneficial for the processability of polymers in extrusion printing processes. In fact, a prominent shear thinning ensures low viscosity values within the nozzle facilitating the extrusion step, while the presence of yield stress results in a rapid increase in the material viscosity at the nozzle exit, where the shear rate drops, thus avoiding material oozing and allowing the deposited material to maintain its shape. It is therefore possible to infer that the composite has enhanced 3D printability compared to the starting PP COPO.

### 3.2. Thermal Characterization

To evaluate the content of crystallinity, which is directly correlated to the volumetric shrinkage of the material, DSC analyses were performed on the 3D-printed material (Figure S1).

From the results in Table 2, it is evident that by adding the nanoclays the polymer crystallization is promoted, with an increase in the crystallization temperature (T_c_), which indicates a faster crystallization of the composite. A decrease in the melting temperature (T_m_) is instead an indication of the formation of a less ordered structure. The higher content of crystallinity in the presence of nanoclays can be explained considering that the fillers act as nucleating agents and induce crystallization of the PP matrix [43]. Similarly, Salavati et al. [44] observed an increase in both the crystallization temperature and the degree of crystallinity upon incorporation of Cloisite20A into neat PP. The observed increase in crystallinity was, however, almost negligible and did not adversely affect the 3D printability of the polymer.

### 3.3. Filament Making Optimization

After the preliminary rheological and thermal characterization of the composite, the pellets obtained through melt compounding in the twin-screw extruder were fed to the Felfil Evo instrument in order to obtain a filament with the appropriate features needed to be 3D printable. Theoretically, an ideal filament should have constant diameter equal to 1.75 ± 0.1 mm, circular cross-section and a perfectly smooth surface. Irregularities in the filament can in fact cause several issues (such as blocks of the pulling system or nozzle clogging) during the printing stage.

The optimization of the extrusion process, in order to achieve a regular filament, occurred by changing three main parameters, namely the extrusion temperature, screw speed and power of the cooling fans. Furthermore, another variable taken into account when optimizing the process was the placement of the fans, in order to optimize the cooling stage and therefore limit the formation of an oval cross-section.

For both the pristine PP COPO and PP COPO/C20A filaments, the optimization of the parameters started from the selection of the temperature and then by modifying the screw speed, power and position of the fans to obtain the required characteristics.

The choice of the correct extrusion temperature is the first fundamental step for obtaining filaments suitable for 3D printing. In fact, a temperature that is too low leads to high viscosity values and these do not allow the extruded filament to stretch sufficiently and reach the desired diameter. On the contrary, a temperature that is too high leads to low viscosity values and therefore to a filament that is not be able to self-sustain when exiting the extruder and which will therefore deform under the action of its own weight.

Regarding the screw speed, low values of 2 and 3 rpm (the maximum speed allowed is 9 rpm) were used, since it was observed that a high speed leads to an excessive amount of material exiting from the nozzle, avoiding regular control on the filament diameter.

Finally, the power and the position of the cooling fan array was optimized. Both of these parameters have a great influence on the shape of the filament section. Regarding the fan speed (maximum value of 255) it was observed that values greater than 200 (approximately 80% of the maximum fan speed) lead to a too powerful air flow and therefore to an oscillation of the filament with subsequent loss of control on the diameter, size and shape. Moreover, it was evident how placing the fan array closer to the extruder head allows one to obtain a more circular section.

The optimized process parameters that have led to filaments with the required characteristics are reported in Table 3.

**Figure 3:**
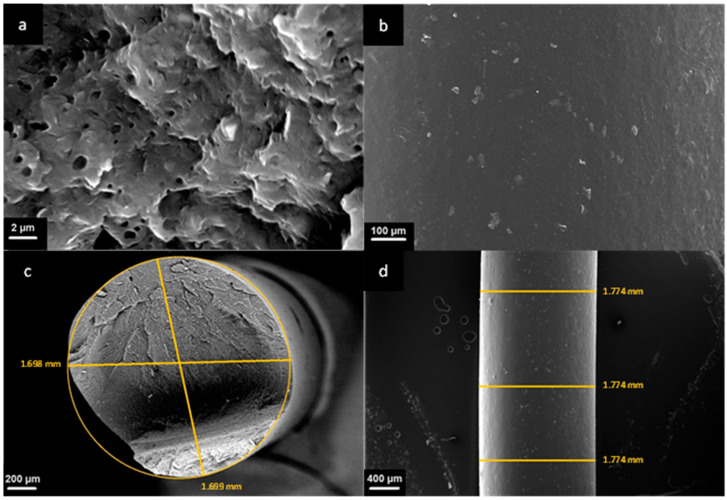
SEM images of the section and the surface of the PP COPO/C20A filament: (**a**) magnification 10,000×, (**b**) magnification 250×, (**c**) magnification 100× and (**d**) magnification 50×.

To evaluate the morphology of the prepared nanocomposite and the superficial quality of our produced filament, SEM analyses were carried out. Figure 3a shows the SEM micrograph (magnification 10,000×) of the fracture section of the PP COPO/C20A filament. Small aggregates of the embedded Cloisite20A can be observed, but considering their sub-micrometric dimensions, it is possible to infer a good dispersion and distribution of the filler lamellae in the polymer matrix. Moreover, at lower magnifications, these aggregates are no longer visible, thus proving, again, the good distribution and dispersion of the nanoclays in the PP matrix. Additionally, the presence of Cloisite20A was also confirmed by the Energy Dispersive X-ray Spectroscopy (EDS), from which the presence of 2.5% Al, 5.9% Si and 0.4% Mg was detected.

From the same micrograph, numerous voids and whiter parts with a roughly spherical shape with average dimensions of about 1 µm can also be observed. These inclusions can be related to the presence of polyethylene or ethylene propylene rubber particles, usually embedded in PP heterophasic copolymers.

From the SEM micrograph in Figure 3b, referring to the external surface of the filament, it is possible to observe that the surface of the filament appears smooth with no evident defects. Concerning the section of the filament, from Figure 3c, it appears to be almost perfectly circular, with two measured diameters of 1699 mm and 1698 mm, respectively. Finally, the external diameter measured in three different points (Figure 3d) is perfectly constant and equal to 1774 mm and therefore is within the range of values compatible with the 3D printer.

### 3.4. Three-Dimensional Printing Optimization

Concerning the 3D-printing process, parameter optimization trials were carried out. As already explained, one of the major issues when 3D printing PP is its poor adhesion on any material different from PP itself. This is why the use of both a PP printing bed and a 3D-printing adhesive was imperative for the optimization of the 3D-printing process. Most of the process parameters, as already described in Section 2.3, were kept unchanged during the whole optimization process, except the extrusion temperature. When printing the PP COPO/C20A samples, four trials, summarized in Table 4, were conducted. When using an extrusion temperature of 260 °C and the printing adhesive, good adhesion of the printed object was achieved. The same parameters were also used for printing the specimens of pristine PP COPO. In this case, however, there was a detachment of the corners of the printed objects, thus proving that the addition of the nanoclays helps to reduce the warpage of the printed parts.

Depending on the analysis, the samples were printed with different deposition patterns, meaning the direction or shape by which the fused filament is deposited on the printing plate in the different layers. In particular, the cone calorimeter samples were printed only with a ±45° pattern, while the UL94 samples were printed with both a ±45° and a concentric pattern.

**Figure 4:**
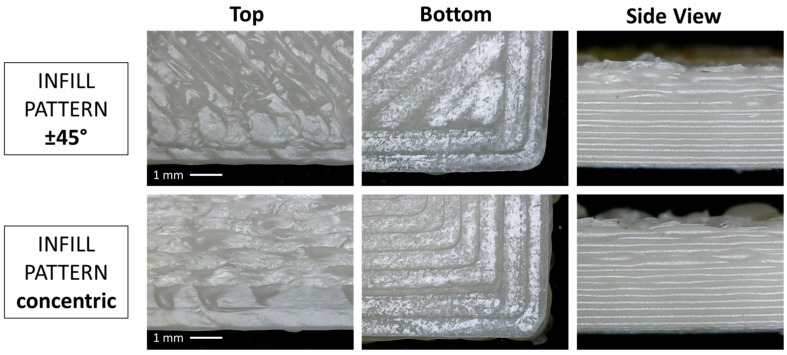
Optical micrographs of UL-94 samples.

From the optical micrographs reported in Figure 4, the difference between the two infill patterns can be observed. In both cases, starting from the left, the top surface, the bottom surface (in contact with the printing platform) and the side view are depicted. From the side view images, it is evident how the accuracy of the deposited layers decreases when increasing the height of the specimen and no obvious differences can be seen between the two kinds of infill pattern.

Furthermore, from the tomography images, the specimens appear to be completely filled without macro voids (Figure S2), while the tensile characterization documented that the FFF-printed samples show relatively high values of toughness (i.e., 558 J/m^3^), suggesting that the optimization of the 3D-printing process conditions prevented the obtainment of brittle samples.

### 3.5. Fire Behavior

#### 3.5.1. Cone Calorimeter

The cone calorimeter tests were carried out on both 3D-printed and compression-molded specimens (CM), considering either pristine PP COPO or PP COPO/C20A. In these analyses, the heat release rate (HRR) [KW/m^2^] over the radiation time was measured and different key parameters, such as the peak of heat release rate (pHRR) [KW/m^2^], total heat released (THR) by the combustion [MJ/m^2^], time to ignition (TTI) [s] and flame out time [s], were evaluated.

**Figure 5:**
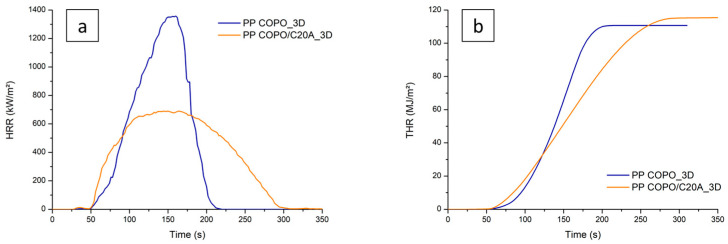
Comparison between cone calorimeter results of PP COPO and PP COPO/C20A 3D-printed samples: (**a**) HRR curves, (**b**) THR curves.

Figure 5a reports the trends of the HRR as a function of time for the 3D-printed specimens with and without C20A. The curve of the PP COPO 3D sample is characterized by a maximum HRR of 1358 KW/m^2^, a TTI of 50 s and a flame out time of 200 s. The presence of the nanoclays leads to a decrease in the value of pHRR to 690 KW/m^2^. As for the TTI, it is 50 s again, but the specimen with Cloisite20A shows a faster initial growth in HRR. Finally, PP COPO/C20A reaches flame out about 90 s after the pristine PP COPO. The improved flame-retardant properties of PP COPO/C20A can be explained as follows: as widely reported in the literature [45,46,47,48,49], the thermal degradation of the organomodifier leads to the formation of acidic sites on the silicate layers. All these catalytic active sites can accept single electrons from donor molecules and form free radicals. These active sites can then catalyze the formation of a protective coat-like char on the nanocomposite that acts as both a barrier to the mass transport of the degradation products and a thermal barrier, preventing additional exposure of the polymer to heat and oxygen. Moreover, the active sites can catalyze the dehydrogenation and crosslinking of polymer chains. Consequently, the thermal–oxidative stability is increased and the pHRR is decreased. A minor contribution to this is also provided by the barrier created through the ablative reassembly of the silicate layers on the polymer surface.

The higher initial HRR can be explained by the fact that the active sites on the layered silicates and acidic sites created by the decomposition of the organomodifier cannot only catalyze the dehydrogenation, crosslinking and charring of the nanocomposite but also the decomposition of the polymer matrix, thus leading to an initial faster growth in HRR.

From the curves of THR as a function of time in Figure 5b, it is possible to observe that the PP COPO_3D sample shows a THR of 111 MJ/m^2^, while the PP COPO/C20A_3D sample shows a slightly higher THR value of 115 MJ/m^2^. However, this last specimen shows a lower slope, indicating that it is characterized by a slower combustion rate and therefore proving that the presence of the nanoclays leads to better flame-retardant properties.

**Figure 6:**
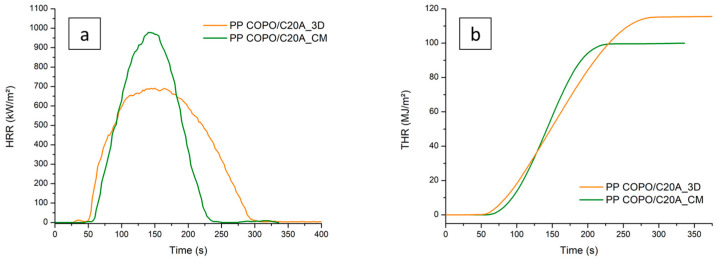
Comparison between cone calorimeter results of PP COPO/C20A 3D-printed and compression-molded samples: (**a**) HRR curves, (**b**) THR curves.

Far more interesting results can be observed in Figure 6a, where it is clear how the processing method strongly influences the final combustion behavior of the material. First of all, it can be seen that the 3D-printed sample has a shorter ignition time (50 s) than the compression-molded sample (58 s). This is probably caused by the higher surface roughness of the 3D-printed specimen, as already observed by Y. Guo et al. [33]. As for the flame out time, the 3D-printed sample shows a delay of flame out time of about 60 s compared to the compression-molded one. Finally, the maximum peak of HRR of the 3D-printed specimen is significantly lower (from 978 KW/m^2^ to 690 KW/m^2^), thus demonstrating that the FFF processing allows one to obtain improved flame-retardant properties compared to CM.

The enhanced behavior of the 3D-printed specimen is also confirmed by the THR curves in Figure 6b. In fact, the FFF-produced specimen shows a curve of lower slope and therefore is characterized by a slower combustion compared to the CM one. It is also evident how the PP COPO/C20A_3D sample shows a higher THR value (115.4 KW/m^2^) than PP COPO/C20A_CM (99.9 KW/m^2^), but this can probably be explained by the fact that the 3D-printed specimen had a higher initial weight (7.25 g) compared to the CM one (6.25 g).

**Figure 7:**
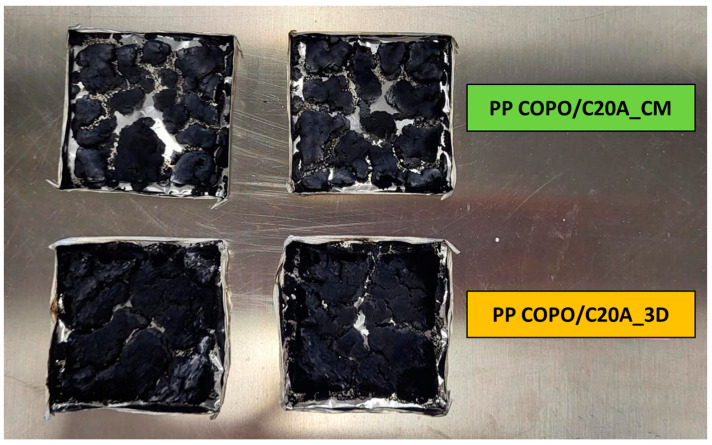
Char residue from cone calorimeter tests of compression molded (**top**) and 3D-printed (**bottom**) PP COPO/C20A samples.

The improved performance of the 3D-printed specimen can be correlated to and explained by observing the carbonaceous residues collected at the end of the cone calorimeter tests. In Figure 7, it is possible to observe that the char formed by the CM specimen is composed of several “islands”, while that of the 3D-printed specimen is more compact and characterized by fewer cracks. The formation of a more compact char can likely be attributed to two different phenomena. Firstly, the shear stress experienced by the material during the passage through the printing nozzle in the FFF process could favor some evolution of the composite microstructure, promoting the achievement of a better dispersion of the nanoclays’ lamellae into the polymer matrix [31]. On the other hand, it should be considered that, due to the layer-by-layer approach for the construction of the FFF sample, the 3D-printing process allows for a much more uniform concentration of nanoclays throughout the specimen. In fact, this process consists of the progressive deposition of single layers, which are all characterized by a homogeneous composition (i.e., each layer contains 5 wt% of nanoclays). Therefore, this feature ensures that the desired amount of nanoclays is present even in the layers on the top of the specimen (which are closer to the radiative source in the cone calorimeter).

Both these phenomena guarantee the presence of a more continuous layer of platelets of C20A in the layer constituting the radiated surface in the cone calorimeter, which, in turn, enables the formation of a more compact and resistant char layer with a better barrier effect on both volatile products and oxygen, hence delaying the thermal–oxidative degradation and decreasing the maximum peak of the HRR. These are very promising and new results; in other similar research works [33,34,50], the specimens printed using FDM techniques have always shown comparable or slightly worst properties to those of compression-molded samples and no significant improvement in the flame-retardant properties has been observed.

#### 3.5.2. Horizontal UL-94

The UL-94 horizontal burning test has been carried out on PP COPO/C20A compression-molded samples and 3D-printed samples with two different infill patterns, a concentric and a ±45° pattern. From the obtained results (Table 5), it is clear how both the production method and the infill pattern influence the burn rate. None of the CM specimens passed the test, while almost all the 3D-printed samples did and took an average of 20 to 30 s longer to burn compared to the CM ones. Comparing the behavior of the 3D-printed specimens with different filling patterns, the deposition of the material in a concentric pattern led to slightly worse results than the ±45° pattern. This demonstrates that the infill pattern is a parameter that deeply influences the final properties of the 3D-printed object [51,52]. In this case, the presence of continuous filaments deposited parallel to the direction of flame propagation in the concentric infill promoted the propagation of the flame itself, while the arrangement of the filaments at ±45° helped to block it.

## 4. Conclusions

A PP-based nanocomposite containing 5 wt% of nanoclays and 3 wt% of PP-g-MA was produced via melt compounding with the aim of developing a filament with flame-retardant properties suitable for FFF techniques. Additionally, the fire-retardant properties of the samples obtained through the 3D-printing FFF process and conventional compression molding were compared.

To investigate the processability of the composite, and in particular its 3D printability, rheological tests were performed and, from the complex viscosity curves, it was observed that the introduction of the nanofiller leads to an amplification of the non-Newtonian behavior of the polymer matrix. Consequently, the composite shows better 3D printability than the starting PP COPO. It was, therefore, possible to extrude the compound with a single-screw extruder and obtain a smooth filament with a constant diameter and circular section. The morphological characterization by SEM also showed that the filler was well dispersed and distributed in the polymer matrix.

The results from cone calorimeter tests documented that the addition of Cloisite20A greatly improved the combustion behavior of PP COPO. Moreover, the 3D-printed samples exhibited slower combustion and significantly lower HRR peak compared to the compression-molded sample. This indicates that 3D printing improves the exfoliation and dispersion of the platelets of nanoclays in the polymer matrix and, most importantly, guarantees a uniform concentration of Cloisite20A in the surface layers of the specimens, resulting in the formation of a more compact char layer with better barrier properties. The superior properties of the 3D-printed samples were also observed in UL94 horizontal burning tests. More specifically, the ±45 patterned 3D-printed samples exhibited a slower burn rate compared to the 0–90 patterned 3D-printed samples, thus proving that the infill pattern used during the printing process can influence the final properties of the 3D-printed object.

In all, this study demonstrated that it is possible to develop and use functionalized polypropylene filaments for 3D printing, and that 3D printing can yield comparable or even superior properties to traditional compression-molding processes.

## Figures and Tables

**Table 1:** Fitting parameters from the Carreau modified model.

Sample	$\eta_{0}$ [Pa]	$\sigma_{0}$ [Pa]
PP COPO	686	2.5
PP COPO/C20A	1110	606

**Table 2:** Scanning calorimetry data of the materials.

Sample	Filler Content [wt%]	T_c_ [°C]	T_m_ [°C]	ΔH_c_ [J/g]	ΔH_m_ [J/g]	χ_c_ [%]
PP COPO	0	113	172	89	61	29
PP COPO/C20A	0.05	117	169	80	64	32

**Table 3:** Optimized parameters for the filaments’ extrusion process.

Material	T_extr_ (°C)	Screw Speed (rpm)	Fan
PP COPO	190	3	180
PP COPO/C20A	190	2	170

**Table 4:** Process parameters and results of 3D-printing trials.

Trial No.	Extrusion Temperature	Printing Adhesive	Result
1	210 °C	No	Detachment
2	210 °C	Yes	Detachment
3	230 °C	Yes	Detachment
4	260 °C	Yes	Good Adhesion

**Table 5:** Results of UL-94 horizontal burning tests. ✕ = fail, ~ = pass the test to the borderline; ✓ = pass the test.

Sample	Average Time [s]	Average Burn Rate [mm/min]	Passed
PP COPO/C20A_CM	91 ± 3	49 ± 2	✕
PP COPO/C20A_3D concentric	113 ± 5	40 ± 2	~
PP COPO/C20A_3D ±45	122 ± 10	37 ± 3	✓

## Data Availability

Data are contained within the article and Supplementary Materials.

## Supplementary Materials

The following supporting information can be downloaded at: https://www.mdpi.com/article/10.3390/polym16060858/s1, Figure S1: DSC traces of the 3Dprinted materials: (A) PP COPO and (B) PP COPO/C20A; Figure S2: X-ray computed tomography images of (a,b) the section and (c) surface of a PP COPO/C20A 3D-printed specimen.
