# Supplementary material for: Development of a Polypropylene-Based Material with Flame-Retardant Properties for 3D Printing
Source: Polymers (Basel). 2024 Mar 21;16(6):858. doi: 10.3390/polym16060858 (PMC10974235; doi:10.3390/polym16060858)
Supplement: Supplementary file 1 [file polymers-16-00858-s001.zip › polymers-2889772-supplementary.pdf]

Supporting Information

Development of a Polypropylene-Based Material with Flame-Retardant Properties for 3D Printing

Eleonora Lorenzi [1], Rossella Arrigo [1,2] and Alberto Frache [1,2,*]

[1] Department of Applied Science and Technology, Politecnico di Torino, Viale Teresa Michel 5, 15121 Alessandria, Italy; eleonora.lorenzi@polito.it (E.L.); rossella.arrigo@polito.it (R.A.)
[2] Local INSTM Unit, 15121 Alessandria, Italy
* Correspondence: alberto.frache@polito.it

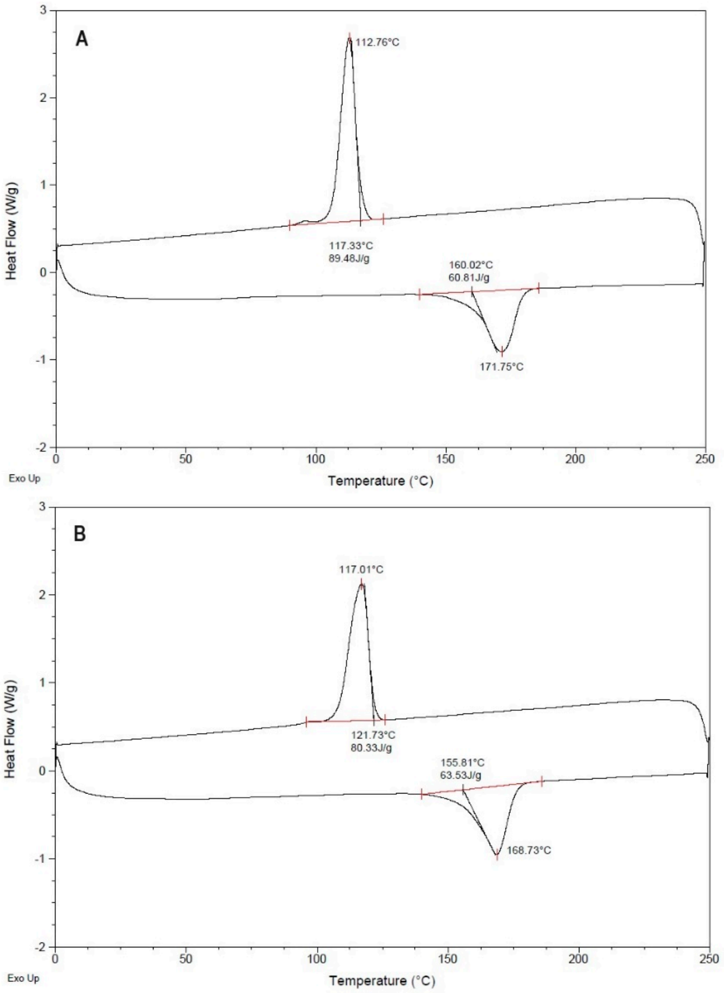

Figure S1. DSC traces of the 3D printed materials: (A) PP COPO and (B) PP COPO/C20A.

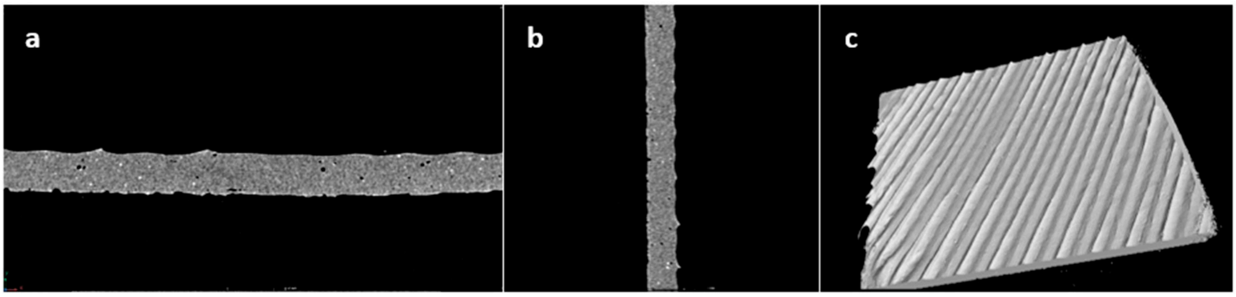

Figure S2. X-ray computed tomography images of (a,b) the section and (c) surface of a PP COPO/C20A 3D printed specimen.
